# A Looking-Glass of Non-Coding RNAs in Oral Cancer

**DOI:** 10.3390/ijms18122620

**Published:** 2017-12-05

**Authors:** Alexandra Iulia Irimie, Cornelia Braicu, Laura Sonea, Alina Andreea Zimta, Roxana Cojocneanu-Petric, Konstantin Tonchev, Nikolay Mehterov, Diana Diudea, Smaranda Buduru, Ioana Berindan-Neagoe

**Affiliations:** 1 Department of Prosthetic dentistry and Dental materials, Division Dental Propaedeutics, Aesthetic, “Iuliu Hatieganu” University of Medicine and Pharmacy, Cluj-Napoca, 23 Marinescu Street, 40015 Cluj-Napoca, Romania; irimie.alexandra@umfcluj.ro (A.I.I.); ddudea@umfcluj.ro (D.D.); 2 Research Center for Functional Genomics and Translational Medicine, “Iuliu Hatieganu” University of Medicine and Pharmacy, 23 Marinescu Street, 40015 Cluj-Napoca, Romania; cornelia.braicu@umfcluj.ro (C.B.); cojocneanur@gmail.com (R.C.-P.); ioananeagoe29@gmail.com (I.B.-N.); 3 MEDFUTURE-Research Center for Advanced Medicine, University of Medicine and Pharmacy Iuliu-Hatieganu, 23 Marinescu Street, 40015 Cluj-Napoca, Romania; laura.sonea16@gmail.com (L.S.); andreea.zimta@umfcluj.ro (A.A.Z.); 4 Department of Maxillofacial Surgery, Medical University, 3 Hristo Botev Blvd, 4002 Plovdiv, Bulgaria; k_tonchev@yahoo.com; 5 Clinic of Maxillofacial Surgery, University Hospital “St. George”, 66 Peshtersko Shosse Blvd, 4002 Plovdiv, Bulgaria; 6 Department of Medical Biology, Medical University Plovdiv, 15-А Vasil Aprilov Bul, 4002 Plovdiv, Bulgaria; ni_ki82@abv.bg; 7 Prosthetics and Dental materials, Faculty of Dental Medicine, “Iuliu Hatieganu” University of Medicine and Pharmacy, Cluj-Napoca, 32 Clinicilor Street, 400006 Cluj-Napoca, Romania; 8 Department of Functional Genomics and Experimental Pathology, The Oncology Institute “Prof. Dr. Ion Chiricuta”, Republicii 34th street, 400015 Cluj-Napoca, Romania

**Keywords:** oral cancer, non-coding RNA, miRNA, lncRNA, piRNA, circRNA

## Abstract

Oral cancer is a multifactorial pathology and is characterized by the lack of efficient treatment and accurate diagnostic tools. This is mainly due the late diagnosis; therefore, reliable biomarkers for the timely detection of the disease and patient stratification are required. Non-coding RNAs (ncRNAs) are key elements in the physiological and pathological processes of various cancers, which is also reflected in oral cancer development and progression. A better understanding of their role could give a more thorough perspective on the future treatment options for this cancer type. This review offers a glimpse into the ncRNA involvement in oral cancer, which can help the medical community tap into the world of ncRNAs and lay the ground for more powerful diagnostic, prognostic and treatment tools for oral cancer that will ultimately help build a brighter future for these patients.

## 1. Introduction

Oral cancer is a form of head and neck cancer, 90% of which consists of oral squamous cell carcinomas (OSCC) [1]. Approximately 300,000 new cases of tumors located in the oral cavity and on the lip are diagnosed each year, for both sexes, on a global scale. Oral cancer is a type of cancer that affects mostly men, the male to female incidence ratio being 2.7; as for the mortality rate, males are again the most affected, the ratio value being 3.8. The pharynx is the most predisposed organ, the tongue ranks second, while the rest of the mouth is the third most common location for oral cancer [2].

Despite the great progresses in cancer diagnosis and therapy, the number of annual deaths due to this malignancy has remained around the same value over the last three decades [3]. The late diagnosis caused by the asymptomatic nature of oral squamous-cell carcinomas in its early stages, despite its accessible location, and the occult initial symptoms, can be easily attributed by patients to trivial causes or overlooked by healthcare providers [4].

Cancer, including OSCC, is regarded as a multifactorial disease, thought to be the result of various genetic modifications that induce the activation of oncogenes and silencing of tumor suppressor genes. Nevertheless, there is increased evidence that deregulated epigenetic mechanisms in association with genetic alterations play a compelling role in the development and progression of human cancers [5].

The complex etiology of OSCC comprises both intrinsic and extrinsic factors. In addition to the two major risk factors associated with OSCC, namely habitual tobacco and alcohol consumption, it is now broadly accepted that chronic inflammation, betel quid chewing, viral infections (HPV) and genetic predisposition are also important players in its pathogenesis. Chronic tobacco and alcohol consumption act synergistically in the development of oral cancer, while HPV infection operates independently, and is considered to have a greater role in oropharyngeal carcinogenesis [5,6,7]. HPV-positive oropharyngeal squamous-cell carcinomas (OPSCC) were revealed as a distinct clinical entity when compared to HPV-negative tumors, particularly in regard to survival rates and response to treatment. Patients with HPV-related tumors have better survival chances and improved prognosis, indicating that a more exhaustive knowledge of these distinctions would aid in the discovery of novel therapeutic approaches for both HPV-negative and positive tumors [8], being characterized by specific non-coding RNAs (ncRNAs) transcripts related to the presence of viral cargo that may affect patient prognostic [9]. All of these components modulate coding and non-coding genes, with impact in early carcinogenic events like OSCC tumorigenesis, but also in distant metastasis formation [10].

ncRNAs are key elements in the physiological and pathological processes of various cancers, which is also reflected in oral cancer development [10]. Several distinct classes of small ncRNAs vary according to their biogenesis mechanisms (structure of their precursors), genomic organization, function, and protein binding-partner [11], but the two main classes of ncRNAs with proven roles in oral cancer are divided, according to their size, into small ncRNA and lncRNAs.

Some ncRNAs are typically obtained from various types of large RNA precursors that are cleaved by RNase III family enzymes (particularly RNAse III Dicer and RNAse III Drosha). The main classes of small ncRNAs include: microRNAs (miRNAs), short interfering RNAs (siRNAs), and PIWI-interacting RNAs (piRNAs), as well as small nucleolar RNAs (snoRNA), small nuclear RNA (snRNA) and repeat-associated RNAs (rasiRNAs). In addition to these, there are most likely other species of small ncRNAs, not yet deciphered and characterized [12]. On the other hand, when referring to the longer ncRNA species such as circular RNAs or long non-coding RNAs (lncRNAs), one of their common characteristics is that their lengths exceed 200 nucleotides [13,14].

**MicroRNAs** (**miRNAs**) and **short interfering RNA** (**siRNA**) are a group of short, single stranded, 20–22 nucleotide RNA molecules, with roles in gene expression regulation [15]. Their biogenesis starts with the transcription of miRNA genes by RNA Polymerase II into pri-miRNAs, which have a stem-loop structure. Next, the pri-miRNA is processed in the nucleus by the Nuclear Microprocessor Complex, which includes RNAse III Drosha and its co-factor, the DGCR8 heterodimer, the result being a miRNA precursor known as pre-miRNA. The pre-miRNA is exported to the cytoplasm with the help of Exportin-5, in an energy-dependent manner. In the cytoplasm, it is further processed by RNAse III Dicer and forms a mature miRNA duplex. At this point, the double-stranded miRNA is incorporated into the RISC (RNA-induced silencing) complex, where the mature strand is retained, while the complementary one is discarded, resulting a fully functional miRNA [16,17,18,19]. MiRNAs bind to their target mRNAs generally at the 3′ untranslated region (3′UTR), where the miRNA recognition elements are located [20]. If the sequence complementarity between the miRNA and its target mRNA is faultless, it will lead to endonucleolytic cleavage and mRNA degradation via the RISC complex. However, this event seldom occurs, since miRNAs have partial complementarity with the target mRNA, which leads to translational silencing of the mRNA [21].

**Figure 1:**
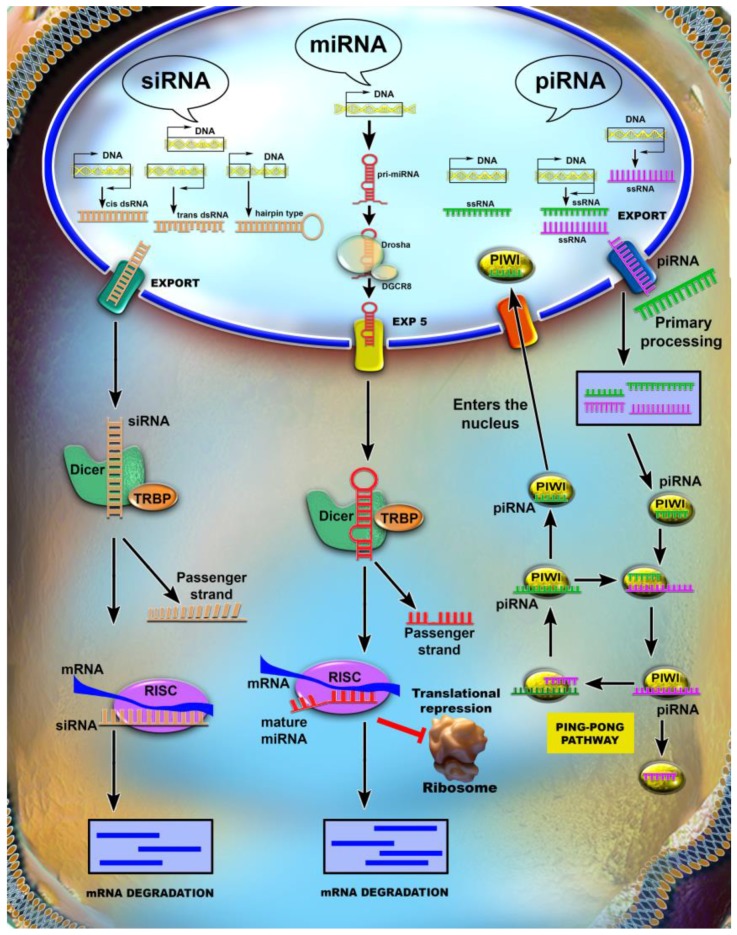
Small non-coding RNAs (ncRNAs) biogenesis. The ncRNAs are transcribed, but not translated. The endogenous siRNA (small interfering RNA (siRNA) can be transcribed from both strands of the DNA, and it can have three double stranded primary forms: *cis*, *trans* and hairpin. The nuclear pore for the export of siRNA is still unknown. In the cytoplasm, the primary form of siRNA is processed by a complex of protein called Dicer into a single-stranded RNA. The main stand in kept and the passenger stand is degraded. siRNA forms a complex with the RISC proteins and, by binding with full complementarity to the mRNA, it inhibits its translation. The microRNA (miRNA) is transcribed only from one strand of the DNA into a primary form with multiple hairpin structure. The primary miRNA is cleaved by the protein complex Drosha and it resulted into a single hairpin structure, called the pre-miRNA that is exported into the cytoplasm via the Exportin 5 nuclear pore. In the cytoplasm, it is processed again by the protein complex Drosha into the mature miRNA with only one strand. The miRNA is then loaded into the RISC complex and binds through short regions to the mRNA. The piwi interacting RNAs are different from the other two classes of small ncRNAs. The piwi-interacting RNAs (piRNAs) are transcribed from both strands of the DNA and are directly transcribed into single-stranded form. The primary form can be loaded onto piwi proteins and induce gene silencing in the nucleus, otherwise the piRNA is exported into the cytoplasm, more precisely in the mitochondria, where it is cut into shorter fragments. The piRNA targets the transposons by entering the ping-pong pathway during which the transposon sequence is silenced and the piRNA is amplified. The rectangles framing the DNA signify the region being transcribed, while the arrows indicate the direction of the transcription. The other arrows stand for the proceeding to the next step in the processing of siRNA/miRNA or piRNA, meaning: transcription, cleavage into smaller transcripts, exportation into the cytoplasm, the interaction with mRNA interaction either during the Ping-Pong pathway in the case of piRNA, or RISC-mediated the case of siRNA and miRNA. The red lines illustrate the repression of an interaction.

**Piwi interacting RNA** (**piRNA**). From their 26–31 nucleotide length, to their specific interaction with piwi proteins, these RNAs separate themselves by several characteristics, among which the fact that they are transcribed, from repetitive sequences in the genome, into single-stranded precursors that bind to piwi proteins and will guide them to endogenous transposable elements, a well-known cause of genetic instability. The piwi proteins belong to the Argonaute family and appear to be highly conserved through evolution [22,23]. The biogenesis mechanisms for the main short ncRNAs are summarized in Figure 1.

**Long non-coding RNAs** (**lncRNAs**) represent a subclass of non-coding RNA transcripts, over 200 nucleotides in size [24]. They comprise a heterogeneous group, and at the same time an abundant constituent of the transcriptome [14,25]. Although most research in this field has so far focused on miRNAs, lncRNAs seem to be equal if not more important participants in the human molecular processes. lncRNAs are transcribed by RNA polymerase II [24], and cannot be exclusively attributed to strictly defined biochemical categories. The lncRNAs are capped at the 5′ terminal region and are frequently spliced and polyadenylated [26,27]. They can originate from various loci on the DNA, from the inter- or intragenic regions to specific chromosomal parts such as telomeres. The primary transcripts can have sense, antisense, bidirectional or overlapping orientations with respect to protein-coding genes [28].

### 1.1. MiRNA Deregulation in Oral Carcinogenesis

MiRNAs are key players in malignant transformation [29]. The miRNA expression profile is different in tumor tissues compared to normal tissue, and also with particular characteristics in distinct tumor types [25]. MiRNAs may participate in tumorigenesis by functioning either as oncogenes or as tumor suppressors [30,31,32,33]. Each miRNA can regulate an average of 100–200 target genes [34,35]. Since they are involved in basically all biological processes, aberrant miRNA expression can trigger the initiation of numerous diseases, including cancer [36].

**Figure 2:**
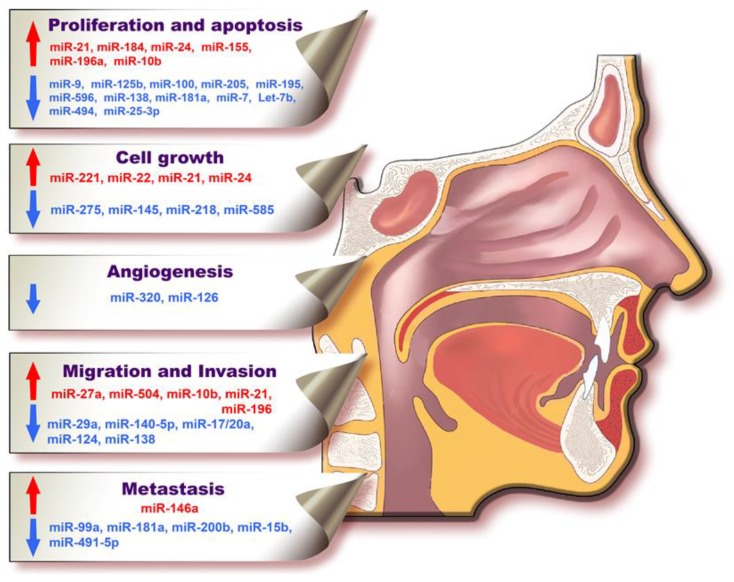
MiRNAs involved in oral cancer hallmarks: proliferation and apoptosis, cell growth, angiogenesis, migration, invasion, and metastasis. In order to understand the complex functions of microRNAs in oral cancer, their involvement was categorized according to the hallmarks of cancer in which they are implicated. Cancer cells evade apoptosis and proliferate continuously. The cells secrete endogenous growth factors that allow them to evade from allogenic signals. In order for the tumor cells to be provided with sufficient nutrients, angiogenesis is stimulated locally. After a certain period of time, cancer cells change their phenotype, enter into the blood stream and invade nearby tissue or metastasize in distant organs. The microRNAs written in red are upregulated in oral cancer and sustain all of the above-mentioned hallmark processes, whereas the microRNAs written in blue are downregulated in oral cancer and are opposing the hallmark processes.

MiRNAs undergo transcriptional and post-transcriptional regulation themselves, affecting key cellular processes. Because of their essential roles in various biological processes, they are actively involved in the maintenance of genomic integrity and suitable cell fate, acting as signal transducer regulators [37,38,39]. Tumor cells developed a set of strategies necessary for their survival and proliferation [40]. In oral cancer, as well as in other cancer types, miRNAs participate in cancer hallmarks [41]. Information related to oral carcinogenesis is summarized in Figure 2.

### 1.2. MiRNAs Involved in Proliferation and Apoptosis in Oral Cancer

A great number of miRNAs are discovered as key participants in tumorigenesis, acting either as tumor suppressors or as oncogenes (oncomiRs). Among them, miR-21 has a pivotal role as an oncomiR by participating in cell proliferation and apoptosis [42], and found to be deregulated in various tumor types [43]. One study proved that miR-21 is overexpressed in progressive leukoplakia and OSCC, but not in normal mucosa or non-progressive leukoplakia, consequently demonstrating its involvement in OSCC progression [44]. In tongue squamous cell carcinoma (TSCC), which is the most frequent type of OSCC, overexpression of miR-21 was correlated with low expression of two of its target genes, *Tpm1* and *Pten*. It was demonstrated that miR-21 overexpression promotes anchorage-independent growth of OSCC cells, partially by targeting *Tpm1* [45]. Furthermore, Zheng et al. showed that miR-21 downregulates the tumor suppressor *P12^CDK2AP1^* at posttranscriptional level in oral cancer cells, and promotes cell proliferation and invasion *in vitro* [46].

Another miRNA with oncogenic role is miR-184, which is overexpressed in TSCC. MiR-184 acts as an anti-apoptotic factor and a promoter of cell proliferation, via alteration of *c-Myc* expression [47]. Nonetheless, another study detected lower levels of this transcript in tumor cells than in normal mucosa, for the same type of tumor [48]. Further research might be needed to clarify the implications of miR-184 in OSCC. MiR-24 has an increased expression in OSCC [49]. By inhibiting the RNA-binding protein DND1, miR-24 regulates several other downstream elements such as cyclin-dependent kinase inhibitor 1B, which sustains cell proliferation and apoptosis evasion [50]. Another OSCC tumor promoter is miR-155, which targets tumor suppressor gene *Cdc73* (cell division cycle 73) and, via this mechanism, increases cell proliferation and reduces apoptosis. The restoration of *Cdc73* expression by miR-155 inhibition stops tumor growth *in vivo* [51]. Oncogenic miR-196a and miR-10b, involved in several other cancers, exhibit high expression levels in oral cancer, as observed in a recent study. These two miRNAs were not previously linked to any of the head and neck squamous cell carcinomas, and their oncogenic role might be due to the deregulation of cell proliferation control mechanisms in the tongue squamous cell carcinoma cell lines SCC25 and SCC9, and pharynx squamous cell carcinoma cell line FaDu [52].

The expression of these miRNAs is generally higher in normal cells than in the poorly differentiated cancer cells, thus showing that miRNA expression is closely linked to cell differentiation [53]. MiR-9 is downregulated in oral cancer and acts as a tumor suppressor in OSCC, by targeting *Cxcr4*, a protein that contributes to tumorigenesis through the Wnt/β-catenin molecular pathway [54]. Another study, conducted by Minor et al., demonstrates that miR-9 might regulate cell proliferation via *Pten*, an important tumor suppressor in oral cancers [55].

In OSCC derived cell lines and OSCC samples, the downregulated expression of miR-125b was associated with higher proliferation rates. This non-coding RNA might exercise its tumor suppressor role by targeting *Icam2* (intracellular cell adhesion molecule 2) [56]. Downregulation of miR-125b and miR-100 in OSCC tumor samples and cell lines was found to be significantly correlated with enhanced cell multiplication and, thus, might play an essential role in tumor development and progression [57]. At the same time, a negative correlation was observed between miR-125b and p53 expression level, and between *TP53* mutation status and miR-125b [58].

MiR-205 has different expression levels in various types of cancer. In OSCC, miR-205 seems to have an oncogenic role [59,60]. It induces the expression of *IL-24* by binding to its promoter sequence, a mechanism of miRNA activity that needs further studying. What is known so far is the fact that, by inducing the overexpression of miR-205, the intracellular level of the pro-apoptotic cytokine IL-24 increases and that miR-205 has the same sequence in the seed as a part of IL-24 promoter. MiR-205 targets the axis inhibitor protein (*Axin-2*), a protein that functions either as tumor suppressor or as tumor promoter in different types of cancer. A recent study proved that miR-195 had low expression rates in TSCC tumor samples, providing evidence that it might act as a tumor suppressor in this cancer type, by inhibiting *Cyclin D1* and *Bcl-2* expression. Through this mechanism, its antitumor effects appear to be manifested as a reduction of cell viability, inhibition of cell cycle progression and increased apoptosis rates [61].

Another proven tumor suppressor, miR-596 is downregulated in OSCC, leading to the upregulation of its target gene, *Lgals3BP*. Consequently, cell proliferation is increased and apoptosis is evaded in oral cancer primary cell lines, through the activation of ERK1/2 signaling pathway [62].

In TSCC cell lines, miR-138 deregulation was also correlated to increased proliferation. Jiang et al. identified *Gnai2* as a potential target for miR-138, by observing that the transfection of this miRNA in TSCC cells reduced the expression of this gene, resulting in diminished proliferation, cell cycle arrest and apoptosis initiation [63].

MiR-181a is frequently under-expressed in OSCC. Under normal conditions, this non-coding RNA was shown to suppress proliferation and anchorage independent growth ability by ectopic expression in OSCC cell lines. A recent study identified the *K-ras* oncogene as one of its main targets in oral cancer [64]. Jiang et al. found that miR-7 acts as a tumor suppressor by downregulating *Igf1R*, which is associated with reduced *Akt* phosphorylation, inhibition of cell proliferation, cell cycle arrest and increased apoptosis [65].

RNAse III Dicer is an endonuclease needed for miRNA maturation [66]. Let-7 family transcripts have been reported to modulate this enzyme [67]. A study from 2010 revealed that *Dicer* expression is abnormal in oral cancer cells, and that this is connected to the downregulation of let-7b. This mechanism led to cell proliferation in oral cancer cell lines [68]. Two of its potential targets were identified in oral cancer, namely *Igf1R* and *Irs-2* [69]. MiR-494 was described as a tumor suppressor in an OSCC cohort primarily formed of tongue cancers. Downregulation of miR-494 was correlated with high expression of *Hoxa10* and a raise in cell proliferation of oral cancer cells [70]. MiR-25-3p expression is reduced in TSCC, and, as a result, the cell-cycle protein expression profile appears disrupted [71].

### 1.3. MiRNAs Involved in Oral Cancer Cell Growth

Cancer cell growth could be stimulated by the highly transcribed miRNAs (Table 1). MiR-221 and miR-222 might have *p27* and *p57* genes as possible targets [72]. A recent paper shows that *Puma* (p53 upregulated modulator of apoptosis) is a direct target of miR-222, and that the downregulation of miR-222 reduces cell growth and induces apoptosis in oral cancer, probably by the direct upregulation of *Puma* expression [73]. Oncogenic miR-21 is also involved in cell growth during oral carcinogenesis, and is positively correlated with *Stat3* expression. There is evidence that inhibition of *Stat3* produces suppression of miR-21, resulting in the upregulation of *Pten*, *Pdcd4*, and *Timp-3* and, cell growth suppression [74]. miR-21 and miR-203 were correlated with the expression level of p63 [58], while miR-24 is another putative cancer generator with unknown mechanism [49].

Another possible mechanism of cell growth in oral carcinogenesis is the downregulation of oncogenic miRNAs in OSCC. MiR-375 was shown to be strongly under-expressed in T3 and T4 tumors, which implies that its suppression might promote tumor growth [75]. MiR-145 is substantially downregulated in oral cancers when compared to the adjacent normal tissues. When restored to its normal transcription rate, miR-145 targets *c-Myc* and *Cdk6*, hence leading to the inhibition of OSCC cell growth [76,77]. Missing parts of the puzzle could be miR-218 and miR-585, often epigenetically silenced in OSCC [78]. Transfection with these two miRNAs in OSCC cells was shown to reduce cell growth, partly via caspase-mediated apoptosis. Furthermore, the mTOR component *Rictor* is a target of miR-218 and, probably, the overexpression of *Rictor* through silencing of miR-218 leads to the activation of the *Tor-Akt* pathway, ultimately contributing to oral carcinogenesis [78].

### 1.4. MiRNAs Involved in Migration, Invasion, Angiogenesis, and Metastasis in Oral Cancer

Angiogenesis, one of the upmost survival strategies developed by cancer cells, is also sustained through alterations of the miRNA transcription process. MiR-320 was identified as a tumor suppressor transcript in OSCC, and it was stated that it might play a crucial part in repressing tumor angiogenesis by silencing *Nrp1* expression [82]. Silencing of miR-126 also correlates with oral carcinogenesis through the activation of angiogenesis and lymphangiogenesis in oral tumors. *Vegf-A* may be a potential target for this miRNA [83].

Cancer cells show their “dark side” when they begin to invade the surrounding tissue and migrate to distant sites, changing also their miRNAs profile (Table 2). It was shown that the overexpression of miR-27, a *Mcph1* repressor, decreased cell invasion and adherence-independent growth of KB cells in soft agar [84]. MiR-504 overexpression in OSCC leads to the downregulation of its target gene, *Foxp1*, and promotes invasiveness of oral cancer cells [85]. Lu et al. [85] determined a specific miRNA profile of OSCC by using miRNA array screening method. Ten miRNAs were proven to be the most significantly associated with this pathology, of which the most upregulated was miR-10b. Further investigations of the roles of this miRNA demonstrated that it actively takes part in oral carcinogenesis by stimulating migration and invasion [86]. MiR-21 is also involved in promoting migration and invasion in OSCC by targeting and downregulating *Pdcd4* expression, correlated with poor overall survival rates [87]. An association between the upregulation of miR-21 and stimulated cell invasion through the Wnt/β-catenin pathway was found. This miRNA acts by targeting *Dkk2* gene [88]. By binding to the mRNA of *Nme4*, miR-196 causes invasion and migration of cancer cells, thus worsening the prognostic of OSCC [89,90].

When miR-29a production is decreased, tumor cells are able to express MMP2 in the large quantities necessary for their invasion and apoptosis escape [91]. In tongue squamous cell carcinoma, miR-140-5p represses cell migration and invasion by directly targeting *Adam10* [92]. Another tumor migration suppressor is miR-17/20a, via its associated gene *Itgb8*. Knockdown of this gene was correlated with reduced migration in OSCC cells [93]. Hunt et al. demonstrated that miR-124 represses OSCC invasion and migration potential by downregulating the expression of *Itgb1* [94]. By targeting *RhoC* and *Rock2* genes, miR-138 is also involved in cell migration and invasion [95].

Oncogenic miR-146a is correlated with tumorigenesis and metastasis in OSCC. Its oncogenic activity was linked to downregulation of *Irak1, Traf6* and *Numb* expression [96]. MiR-181’s potential oncogenic effect in OSCC may act by initiating migration and enhancing lymph node metastasis [97].

A tumor suppressor microRNA, which was observed to be frequently downregulated in OSCC, is miR-99a. Its repression was correlated with enhanced metastasis potential. miR-99 family contributes to oral cancer tumorigenesis by targeting IGF1R and mTOR signaling pathways [99]. In TSCC cell lines, miR-181a downregulation was associated with higher metastatic potential, possibly via overexpression of its target gene, *Twist1* [100]. MiR-200b and miR-15b are also involved in the development of TSCC, by inhibiting tumor metastasis [98].

Tumor suppressor miR-491-5p exerts its role in repressing OSCC metastasis by targeting *Git1*, which leads to the perturbation of FAK/paxilin and *ERK1*/*2* signaling along with *MMP2*/*9* expression and activity [101].

### 1.5. Piwi-Interacting RNA (piRNA) Effects in Oral Cancer

In comparison with siRNA and miRNA, the number of studies focusing on piRNA is very limited. In the case of head and neck carcinoma tissue, piRNA presented altered expression values when compared to normal tissue, particularly meaning an affluence of these types of non-coding RNAs in cancer cells. The expression pattern for a 41-member Piwi panel was found to differ between HPV-positive and HPV-negative head and neck squamous cell carcinoma (HNSCC) samples, with 11 of them being overexpressed distinctively in HPV16 or HPV18 induced tumors. Of these, 5 were correlated with patient survival rates, namely piR-35953, piR-36984, piR-39592, piR-36715 and piR-30506 [102]. This molecular signature in HPV-positive tumors was related to an unfavorable survival rate [102,103].

Recently, a panel of 13 piRNAs was identified in OSCC related to smoking, from which NONHSAT123636 and NONHSAT113708 are directly correlated with tumor stage, along with NONHSAT067200, which predicts the patient survival rate. PIWIL1 was related to genomic alterations, including in the Tp53 gene [103].

### 1.6. LncRNA Deregulation in Oral Cancer

**Figure 3:**
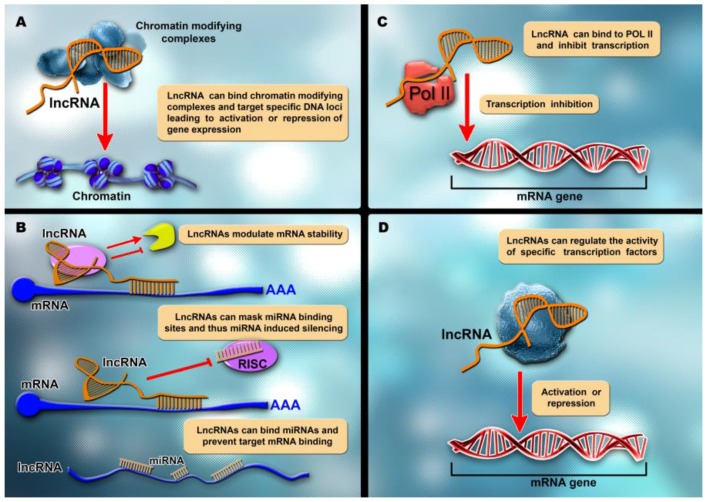
LncRNAs have multiple functions in the cells: (**A**) lncRNAs can change gene transcription by interfering with chromatin state, through the chromatin-binding complex, related to the presence of some specific chromatin modifying factors with enhancer and promoter sequences to enable gene expression; (**B**) lncRNAs can inhibit the translation of mRNA by directly binding to the mRNA, by masking the microRNA binding site; (**C**) lncRNAs interact with polymerase II and inhibit transcription; and (**D**) lncRNAs interfere with transcription by interacting with transcription factors.

LncRNAs have a wide variety of functions, of which the epigenetic regulation of protein-coding genes is one of the main players [104]. They control transcription by chromatin modulation, by acting as scaffolds for chromatin modifying complexes. They also upregulate transcription of enhancers, and can influence epigenetic events via transcription-dependent mechanisms, along with directly influencing the transcription machinery. Aside from regulating all aspects of gene expression, lncRNAs are also involved in the regulation of mRNA processing, protein activity and post-transcriptional control (Figure 3). They can function as scaffolds for higher-order complexes, signaling molecules via exosomes, and vehicles for increased genetic diversity [105]. The tissue specificity of lncRNAs might enable them to be valuable biomarkers and therapeutic agents [41], based on their expression levels or related with the presence or absence of certain mutation [106].

**Figure 4:**
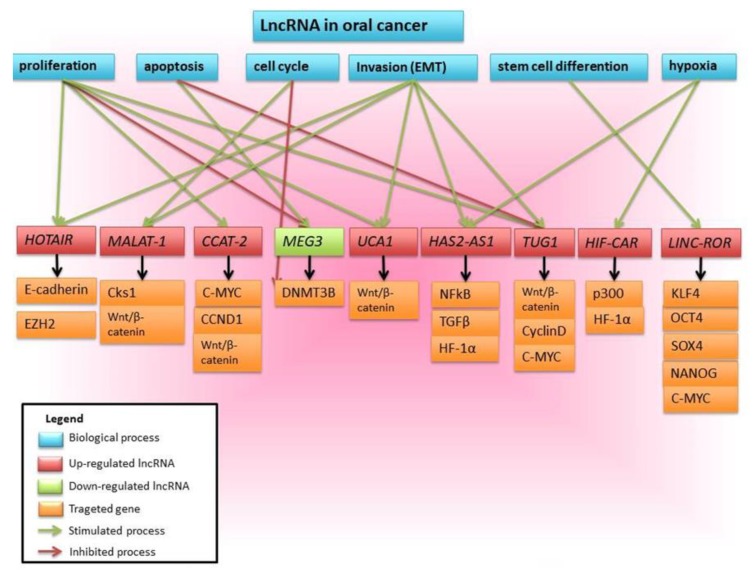
Schematic view of the multiple OSCC biological processes involving lncRNAs and their target genes. The lncRNAs *HOTAIR*, *MALAT-1*, *CCAT-2*, *UCA1*, *HAS2-AS1*, *TUG1*, *HIF-CAR*, and *LINC-ROR* are upregulated and promote oral cancer progression, while the lncRNA *MEG3* is down-regulated with prognostic role.

Similar to miRNAs, lncRNAs were found to act as tumor suppressors or oncogenes in the development and progression of human cancers, offering a new level of complexity to the molecular pathways of carcinogenesis [107]. Since lncRNAs have been known to modulate of a wide variety of biological processes, such as transcriptional regulation or genomic imprinting, they are beginning to be considered central players in the human cancer scene [27]. Hence, one can safely assume that lncRNAs are also associated with oral cancer (Figure 4). Unlike miRNAs, the studies investigating the roles of lncRNAs in oral cancers are still scarce (Table 3).

Gibb et al. [27] were the first to evaluate the lncRNA expression profile for oral mucosa, identifying the expression of 325 lncRNAs in normal tissues, out of which about 60% showed statistically significant deregulations in oral dysplasia. They found that *Neat1* was the most overexpressed in human oral mucosa [108]. The expression of several well-researched lncRNAs from the saliva and tissues from patients with OSCC were associated with cancer. *Hotair*, *Neat1* and *Uca1* were found to be overexpressed, mainly in metastatic tumors, while *Meg-3* expression was downregulated [109]. The expression of *Meg-3* is significantly affected by the degree of DNA methylation, with important role in patient prognostic [110].

*Neat1* (Nuclear Enriched Abundant Transcript), by being up regulated in various cancer tissues, was found to promote tumorigenesis and cancer progression. It inhibits apoptosis and stimulates growth and metastasis [111]. By lowering the normal expression of the tumor suppressor miR-107, *Neat1* causes the laryngeal squamous cell carcinoma cell line Hep-2 to have an increased *CDK6* expression. *Neat1* also induces cell cycle progression in the cancer cells, along with apoptosis resistance and enhanced invasion [112].

The lncRNA *Hotair*’s involvement in human tumorigenesis was widely studied [25]. Aside from modulating the expression of numerous genes, its extensive role still remains poorly understood [113]. However, it was stated that it has substantial impact on proliferation, epithelial-mesenchymal transition and metastasis in various human cancer types [113,114,115,116,117,118]. Tang et al. found an overexpression of *Hotair* in samples from OSCC patients, especially with lymph node metastases [109], these results being in accordance with the aforementioned studies on *Hotair*. In oral cancer, *Hotair* sustains cell proliferation [119], invasion and metastatic processes by targeting *Ezh2* and repressing *E-cadherin* [120]. The important role of *Hotair* as a biomarker is sustained by a recent meta-analysis study [121]. *Hotair* overexpression was related with unfavorable prognostic, advanced tumor stage and the presence of metastases [121]. In the case of *Hotair*, certain genetic alterations (rs920778, uc003opf.1, and rs11752942) were related with head and neck cancer susceptibility [106].

*Malat1*/*Cks1* pathway was connected to OSCC tumor radiosensitivity [122]. Taurine upregulated gene 1 (*Tug1*) was overexpressed in OSCC, correlated with TNM stage and lymph node metastasis [123].

*Uca1* (urothelial cancer associated 1) is a lncRNA that plays a central role in bladder cancer growth, progression and invasion [124,125,126,127]. It was also found to be overexpressed in other cancer types, such as colorectal cancer [128], esophageal squamous cell carcinoma [129], melanoma [130] and breast cancer [131]. The expression of *Uca1* was evaluated for TSCC, the most frequent form of OSCC [132,133]. It was overexpressed and correlated with the migration ability of cancer cells. Considering that *Uca1* levels were higher in advanced TSCC, it was hypothesized that its deregulation primarily occurs in cancer progression and not in its initiation [134]. *Uca1* overexpression is related with OSSC progression via *WNT*/*β-catenin* signaling pathway [135] and was demonstrated to promote metastasis.

Maternally expressed gene 3 (*Meg3*) encodes a lncRNA produced by various normal tissues, which plays the role of tumor suppressor [136,137,138,139]. The loss of this RNA expression causes cell growth and proliferation in human cancers, thus supporting the claim that *Meg3* is a tumor suppressor lncRNA [140]. *Meg3* is among the most substantially underexpressed ncRNAs in cancer [141], leading to apoptosis arrest, cell cycle progression and almost unstoppable proliferation [109,141]. miR-26a and *Meg3* were correlated with cancer progression, having prognostic value for patient stratification [141].

*Ccat2* (colon cancer-associated transcript 2) is an important transcript that was proven to have regulatory effects in several cancer types. *Ccat2* has been proven to mediate the malignant behavior of cells by suppressing *β-catenin*, *Ccnd1*, and *Myc* [142]. The increased level of *Ccat1* appeared to be related to its capacity to sponge miR155-5p and let7b-5p, leading to an unfavorable prognostic [10]. *Has2-As1* was related to the hypoxia-regulated EMT and invasiveness of OSCC [143]. *LncHIFCAR* level is substantially upregulated in OSCC and it was demonstrated to have a crucial role in tumorigenesis [133]. *linc-RoR* was proven to be overexpressed in undifferentiated OSCC, hence having prognostic value [144].

### 1.7. Circular RNAs

Circular RNAs (circRNAs) are circles of ncRNAs with no 5′ polyadenylated tail, having a linkage between the 3′ and the 5′ ends, to form a covalently closed continuous loop. They are transcribed as mRNAs, but in the downstream steps they are processed differently, through alternative mechanisms, such as backsplicing by RNA polymerase II, as *cis* or *trans* forms [151].

Because they are initially mRNA precursors that end up being ncRNAs, circRNAs are believed to indirectly modulate gene expression via miRNA sponging activity, translational repressor or via posttrascriptional regulation activity [151]. CircRNAs entrap the microRNAs by binding to them and thus stopping miRNA silencing, which is why circRNAs are also called miRNA sponges [13,152]. These transcripts are actively involved in the regulation of miRNA activity, which can be applied as therapeutic strategy to target the overexpressed miRNAs.

CircRNA-100290 is upregulated in oral cancer tissue and it induces cancer progression by sponging the miR-29 family members. CircRNA_100290 is correlated with OSCC cells proliferation *in vitro* and *in vivo*. The expression of this circular RNA is correlated with CDK6, a target of miR-29B [153].

### 1.8. Free and Exosome Mediated ncRNA Transfer in Oral Cancer

In recent years, many circulating biomarkers have been assessed, including in oral cancer. These circulating ncRNAs can be used for an improved diagnostic and for monitoring the response to therapy [154]. The research direction is focused on implementing the use of liquid biopsy specimens in common practice for precision medicine, similar to those obtained from tissue biopsy [72]. The main problem related to this process is the lack of standardized methods for sampling, evaluation and particularly for the normalization methods [155]. These will lead to increased power and accuracy of data, and a rapid implementation in clinical practice [154]. The ncRNAs with an altered expression level in tumor tissues and confirmed in different biological fluids (plasma, oral cytology, and saliva) in free form or exosome-trapped fluids can have important roles in oral cancer management.

The comparison among the microRNA profile of tumors, benign tissue, plasma and serum exosomes from patients with TSCC revealed that there were common down-regulated microRNAs in tumors, plasma and serum exosomes (miR-370, miR-139-5p, miR-let-7e, and miR-30c); microRNAs found in tumors and exosomes (miR-22 and miR-145-3p); or a microRNA found only in tumors (miR-516-3p) [156].

Exosomes are nanometer-sized microvesicles involved in cellular communication due to their capacity to transport bioactive molecules (proteins, lipids, and nucleic acids, particularly ncRNAs). Exosomes have the capacity to transfer cargo related to physiological or pathological status [157,158]. These vesicles represent a valuable source in biomarker discovery, due to their cargo, which was connected with cancer progression and distant metastases [159]. Exosomes cargo in oral cancer was related to viral contamination, and thought to affect the microenvironment.

Exosomes can be assessed by non-invasive tests which evaluate biological fluids, including saliva [159]. It was demonstrated that most of the salivary miRNAs are retrieved from exosomes [160], which is of great interest. The ncRNAs with exosomal origin are protected from enzyme degradation, therefore are much more stable than salivary free ncRNAs [159,161]. Because of this, saliva exosomes represent a valuable source of ncRNAs biomarkers.

Oral epithelial cells display several miR-200 family members which can be transported as exosomal cargo to proximal EBV-positive B cells, where they trigger reactivation of inflammatory processes leading to the activation of the cancer phenotype [162]. It was demonstrated that exosomes from hypoxic oral cancer cells deliver miR-21 to normoxic cells, as a premetastatic event [163]. As an example, exosome-trapped miR-1246 was related with increased cell motility and invasion [164].

These facts remain to be further validated by showing how saliva exosomal cargo affects oral cancer progression and how this source of biomarkers can be exploited with the purpose of pursuing personalized care.

## 2. Conclusions and Perspectives

The altered expression of ncRNAs was related to the regulation of key cellular processes. A better comprehension of these molecular mechanisms regulated by ncRNAs and how they control the oral cancer phenotype and its relation with environmental factors can become the basis for developing more effective therapies, and controlling the expression of ncRNAs can be the fundament for oral cancer precision medicine. All this information related to altered ncRNA expression patterns emphasizes the molecular heterogeneity of oral cancer, with noteworthy repercussions on the selection of candidates for the progress of novel targeted therapies or prognostic screening protocols, allowing a personalized treatment.

MicroRNAs are the most well studied ncRNAs in all types of malignancies, and oral cancer is no exception. The various miRNAs, each with its own palette of targeted genes, acts on various signaling pathways that sustain oral cancer hallmarks, such as apoptosis evasion, sustained proliferation, autonomous growth, angiogenesis, invasion and metastasis, which is why microRNAs are commonly regarded as the best candidates for developing new cancer therapies. piRNAs are a less studied class of small non-coding RNAs in all types of pathologies, and their mechanism of action still poses many questions; nevertheless, they have the potential of becoming future cancer biomarkers, considering that they have an altered expression pattern with prognostic value that can be useful for OSCC patient subtype stratification.

LncRNAs are a diverse type of ncRNAs that not only act at the mRNA level, but also interfere with DNA folding and transcription process. Their involvement in oral cancer is very complex, and is encountered on numerous levels, such as proliferation, apoptosis, angiogenesis, reaction to hypoxia, stem cell phenotype maintenance and initiating invasion through the EMT process.

Finally, circular RNAs are stepping forward into the light of ncRNAs involvement in oral cancer, motivated mainly by the fact that they act as miRNA sponges. A certain type of circRNA can entrap a multitude of oncomiRs and allow the transcription of hundreds of tumor suppressor genes, which is why their future as oncological therapy options seems very promising.

The world of ncRNAs remains a subject for a significant number of studies, where an important role will be represented by the free or exosome-trapped lncRNA, not only with prognostic value, but also for monitoring the response to therapy, such as the case of miR-21 secreted by hypoxic tumor core. Hopefully, in the near future, the acquired information will lay the ground for more efficient diagnostic, prognostic and treatment options for oral cancer that will ultimately result in a decline of its mortality rates and a better quality of life for the patients.

## Figures and Tables

**Table 1:** The main altered miRNAs involved in cell growth, proliferation and apoptosis.

Effects	Expression Level	Name	Targets	Tissue Specimens/Cell Line/Animal Model	Methods and Materials	Main Results of the Study	Reference
Cell Proliferation	Down	miR-10b	-	Tissue from OSCC and HNSCC patients Cell lines: SCC25 and SCC9, FaDu compared with primary culture of oral keratinocytes	microarray, qRT-PCR, transfection with siPORT NeoFx reagent (Ambion), immunofluorescence assay, flow cytometry, Western blotting	MiR-10b precursors transfection reduce cell proliferation and cell cycle arrest by targeting the *TP53*, *NOTCH1*, *MYC* and *HRAS* gene	[52]
Apoptosis, cell proliferation	Up	miR-21	*TPM1* *PTEN* *P12CDK2AP1, HIF-1Α*/*HIF-2A*	FFPE specimens from patients, Cell lines: SCC-15, CAL27 SCC9, SCC15, Tca8113 *In vivo*: BALB/c-nu mice	qRT-PCR, microarray, northern blotting, TUNEL assay, Western blotting, MTT assay, colony formation assay, soft agar assay, Annexin V analysis, cytochrome c release, caspase-3 assay, Transwell invasion assay,	miR-21 overexpression was related with and unfavorable prognostic; miR-21 sustains cell proliferation, activate tumorigenesis in hypoxic conditions	[44,45,46,79]
		miR-24	*DND1*	Cell line: UM1, UM2, Cal27, SCC4, SCC1, SCC2, SCC9, SCC15, SCC25, NOK16B	qRT-PCR, Western blotting, dual luciferase reporter assay, MTT assay, Annexin V-FITC apoptosis detection kit, flow cytometry	Regulate cell proliferation and apoptosis	[50]
		miR-155	*Cdc73*	Cell lines: KB, SCC084, SCC131 BALB/c mice	*in silico* identification of miRNA binding sites, plasmid construction, Lipofectamine transfection, qRT-PCR, northern hybridization, Western hybridization, sequencing with ABIprism A310-automated sequencer, LOH analysis at the CDC73 Locus, combined bisulfite restriction analysis, MTT assay, fluorescein active caspase-3 staining kit, soft agar assay, propidium iodide analysis, *in vivo* assay for tumor growth	Cell proliferation and tumorigenesis	[51]
		miR-184	*c-Myc*	Tissue samples from: normal oral mucosa, leukoplakia and oral cancer tissue	qRT-PCR, immunohistochemistry	miR-184 inhibitor reduce cell proliferation and increased apoptosis rate	[47]
		miR-196a	*TP73*, *CDK2*, *AKT1*	Tissue from OSCC and HNSCC patients Cell lines: SCC25 and SCC9, FaDu compared with primary culture of oral keratinocytes	microarray, qRT-PCR, transfection with siPORT NeoFx reagent (Ambion), immunofluorescence assay, flow cytometry, Western blotting	regulation of apoptosis and cell cycle progression	[52]
	Down	Let-7b	*Igf1R*	Cell lines: CAL 27, SCC-25, FaDu, RPMI 2650 Xenografts of Cal27 cell in NOD-SCID mice Cell lines: Tca-8113, Cal-27, primary OSCC and normal tissues *In vivo*: BALB/C nude mice	Western blotting, indirect immunofluorescence, qRT-PCR, Lipofectamine transfection, transfection using X-tremeGENE HP DNA transfection reagent and X-tremeGENE siRNA transfection reagent, reporter plasmids construction, luciferase assays, lentivirus infection, MTT assay, flow cytometry, kinase activity assay kits, colony formation assay, immunoblotting, immunohistochemistry	Regulation of cell proliferation	[67,68,69]
		miR-7	*Igf1R*	Cell lines: UM1 and UM2	Western blotting, dual-luciferase reporter assay, qRT-PCR, MTT assay, flow cytometry	Regulated apoptosis and cell proliferation	[65]
		miR-9	*CXCR4* *PTEN*	Cell line: SCC-4, SCC-9, SCC-25, Tca8113 Xenograft mouse model injected with Tca8113 and SCC-9 human surgical samples of HNSCC tissue	lentiviral delivery of miR-9, XTT assay, flow cytometry, Annexin V affinity assay, QCM invasion assay kit, immunohistochemistry, plasmid construction, luciferase reporter assay, Western blotting MS-PCR, qRT-PCR, transfection using DharmaFect, MTT assay	Restoring the expression level leads to the decreased cell proliferation, colony-formation abilities, cell cycle arrest and stimulated apoptosis.	[55,80]
		miR-25-3p	-	Cell lines: Tca8113	viral infection of Tca8113, MTT assay, qRT-PCR, Western blotting	Restoring the expression level leads to a decreased proliferation and colony-formation ability, along with lower cyclin D mRNA and protein level.	[71]
		miR-100	-	Cell lines from UPCI: SCC collection and NHOK controls	qRT-PCR, FISH with RP11-241D13, Lipofectamine transfection, MTT assay, microarray	decreased proliferation and colony-formation ability, along with lower cyclin D mRNA and protein level and an important altered transcriptomic pattern	[57]
		miR-125b	*ICAM2*	Cell lines: HSC-2, HSC-3, HSC-4, SCC4, HO-1-N-1 and Ca9-22 compared with human normal keratinocytes Cell lines from UPCI: SCC collection and NHOK controls	qRT-PCR, Lipofectamine transfection, construction of reporter plasmids, luciferase reporter assays, cell count in hemocytometer, X-ray irradiation, clonogenic survival assay with crystal violet FISH with RP11-241D13, Lipofectamine transfection, MTT assay, microarray	Prognostic marker, decrease in miR-125b expression was associated with poorer survival	[56,57]
		miR-138	*GNAI2*	Tissue samples from TSCC patients Cell lines: SCC4, SCC9, SCC15, SCC25, Tca8113, UM1 and UM2	microarray, qRT-PCR, Western blotting, dual luciferase reporter assay, MTT assay, apoptosis assay with Annexin FITC conjugate	Regulation of apoptosis and cell proliferation	[63]
		miR-181a	*K-ras*	Primary normal human oral keratinocytes Cell lines: HOK-16B (oral keratinocytes), SCC-4, SCC-9, SCC-15, 1483, Tu-139, and Tu-177	qRT-PCR, transfection using lentiviral vector, MTT assay, anchorage-independent growth in soft agar, Western blotting, K-ras 3′-UTR and miRNA promoter constructs, luciferase assay	Regulate cell proliferation, EMT and invasion	[64]
		miR-195	*Cyclin D1* *Bcl-2*	TSCC samples from patients Cell lines: SCC-15 and CAL27	qRT-PCR, immunohistochemistry, *in situ* hybridization, cell counting kit, fluorescence-activated cell sorting (FACS) by flow cytometry, vector construction, luciferase reporter assay, Western blotting	Regulate cell cycle and apoptosis	[61]
		miR-205	*Axin2* *IL-24*	Cell lines: KB compared with normal keratinocytes NHOK	microarray, qRT-PCR, qPCR, Lipofectamine transfection, MTT assay, DAPI staining fluorescence microscopy, immunoblotting, caspase-3/7 activity assay by using cell-permeable fluorogenic substrate, PhiPhiLux-G1D2, luciferase assays, Annexin V-fluorescein isothiocyanate assay	Restoring miR-205 expression level activates apoptosis via caspase-3/7 and modulated immune response	[59,60]
		miR-494	*Hoxa10*	human OSCCs and normal tongue tissues Cell lines: SCC-25 and CAL 27	qRT-PCR based on the microarray result from a previous study, Lipofectamine transfection, dual luciferase assay	Regulation of cell proliferation	[70]
		miR-596	*Lgals3BP*	Cell line: RT7, Primary OSCCs and normal oral mucosa *In vivo*: SCID mice	qRT-PCR, microarray, transfection with Lipofectamine, Western blotting, luciferase activity assay, immunohistochemistry	Restoration of the expression of miR-596 in OSCC cells *in vitro*, caused increased in cleaved caspase-3, apoptosis and cell cycle arrest in the G1 phase, reduction of tumorigenesis in mice models	[62]
Cell Growth	Up	miR-21	*Stat3*	OSCC tissue samples from patients Cell line: TSCCA and TCA8113 TSCCA xenograft nude mouse model	miRNA detection by in situ hybridization, qRT-PCR, flow cytometry, MTT assay, Transwell assays, luciferase assay, Western blotting, *in vivo* tumor measurement, IHC staining, TUNEL assay	Inhibition the expression level leads increased apoptosis, via inhibition expression of Ki67, Bcl-2 and MMP-2	[74]
		miR-24	-	OSCC tissue samples from patients Cell OSCC cell line: OC3, OECM-1 and SAS OSCC compared with normal human keratinocytes (NHOKs) and 293T cells	qRT-PCR, lentiviral vector construction, Western blotting, trypan blue exclusion assay, Transwell assay	miR mimetic stimulated cell growth and inhibition of p57, unaffected the EMT-related genes or caspase-3	[49]
		miR-221	*p27*	OSCC tissue from patients Cell lines: OECM-1, SAS *In vivo*: nude mice	qRT-PCR, immunohistochemistry, plasmid preparation, lentiviral infection, trypan blue exclusion assay, anchorage e-independent colony formation assay counted by crystal violet staining, Transwell assay, Western blotting, *in vivo* tumorigenesis	miR-221 transfection caused greater cell/tumor growth	[81]
		miR-222	*p57* *Puma*	OSCC tissue from patients Cell lines: OECM-1, SAS *In vivo*: nude mice Cell lines: Tca8113 and UM1	qRT-PCR, immunohistochemistry, introduction of miRNA through plasmid preparation and lentiviral infection, trypan blue exclusion assay, anchorage-independent colony formation assay counted by crystal violet staining, Transwell assay, Western blotting, *in vivo* tumorigenesis Lipofectamine transfection, RT-PCR, Western blotting, immunofluorescence, cell migration assay by 8-μm pore insert, MTT assay, Annexin V analysis	miRNA mimetic caused decreased apoptosis, increasing cell proliferation and migration	[72,73]
	Down	miR-145	*c-Myc* *Cdk6*	OSCC tissue samples from patients Cell line: Tca8113, CAL 27	Oligofectamine transfection, qRT-PCR, Western blotting, MTT assay, colony formation assay, anchorage independent growth assays, flow cytometry, Annexin V analysis, Transwell chamber histological analysis, clinicopathologic factors analysis	Restoring the expression level affect cancer hallmarks, including stimulation of the apoptosis and cell cycle arrest	[76,77]
		miR-218	*Rictor*	RT7, human oral keratinocytes immortalized by TERT and primary OSCC samples-NA and SKN3	miRNA function-based screening, methylation analysis, qRT-PCR, Lipofectamine transfection, immunoblotting, luciferase activity assay	Targets the mTOR, inhibits AKT phosphorylation leading to the regulation of cell proliferation and apoptosis	[78]
		miR-375	-	OSCC tissue samples from patients	microarray, qRT-PCR	miR-375 downregulation was correlated with disease progression and a poorer prognostic. Regulated apoptosis related proteins	[75]
		miR-585	-	RT7, human oral keratinocytes immortalized by TERT and primary OSCC samples-NA and SKN3	miRNA function-based screening, methylation analysis, qRT-PCR, Lipofectamine transfection, immunoblotting, luciferase activity assay	Regulate cell proliferation and apoptosis	[78]

**Table 2:** The main altered miRNAs involved in migration, invasion angiogenesis, and metastasis in oral cancer.

Effects	Expression Level	Name	Targets	Tissue Specimens/Cell Line/Animal Model	Methods and Materials	Main Results of the Study	Reference
Angiogenesis	Up	-	-				-
	Down	miR-126	*VEGF-A*	OSCC tissue samples from patients Cell lines: HSC3 and HSC4 cells	qRT-PCR, methylation-specific PCR, DNA demethylation treatment, Lipofectamine transfection, MTT assay, invasion assay by using modified Boyden chamber, TUNEL assay, immunohistochemistry	miR-126 downregulation activate angiogenesis and lymphangiogenesis; prognostic marker	[83]
		miR-320	*Nrp1*, *HIF-1α*	Tumor tissue and adjacent normal tissue specimens from OSCC patients and human umbilical vein endothelial cells *In vivo*: NOD/SCID mice	*in situ* hybridization, RT-PCR, Western blotting, plasmid construction, Lipofectamine transfection, luciferase reporter assays, NRP1 knockdown through infection by lentivirus containing NRP1 shRNA, Transwell migration assay, Tube formation assay by using human umbilical vein endothelial cells, *in vivo* tumor size measurement	miR-320 precursor/antagonist reduce migration, adhesion and tube formation of vascular endothelial cells; miR-320 is inhibited in hypoxic condition	[82]
Migration and Invasion	Up	miR-10b	-	Cell lines: SCC25, SAS, OECM1, OC3, CGHNC8, CGHNC9 and normal keratinocytes: CGHNK2, CGHNK4, CGK1, CGK5, and CGK6 Plasma samples from OSCC patients	microarray, qRT-PCR, Lipofectamine transfection, colony formation assay by cell strained with crystal violet, *in vitro* wound-healing assay, Matrigel invasion assay, determination of chemo- or radiosensitivity, determination of plasma miRNA, clinical data analysis	Regulate cell migration and invasion; diagnostic marker	[86]
		miR-21	*Pdcd4* *Dkk2*	OSCC tumor samples from patients Cell lines: UT-SCC-15, 20A, 24A, 28, 74A, 87 compared to a normal oral mucosa Cell line cell line: SCC25	QPCR for PDCD4 mRNA levels, immunohistochemistry, plasmid construction, Lipofectamine transfection, Transwell invasion assay, Western blotting, RT-PCR, site-directed mutagenesis, in situ miRNA hybridization, knockdown of miRNA with anti-sense LNA oligomers, transfection by using Oligofectamine reagent, Matrigel invasion chamber, clinical data analysis	Regulate EMT, invasion, migration and metastasis; therapeutic target in oral cancer	[87,88]
		miR-27a	*Mcph1*	Cell line: KB, SCC084, SCC131 *In vivo*: BALB/c nude mice	LOH analysis, sequencing by ABIprism A310-automated sequencer, Western blotting, qRT-PCR, immunohistochemistry, promoter methylation analysis, treatment of cells with 2′-deoxy-5-azacytidine, Lipofectamine transfection, BrdU cell proliferation assay, soft agar colony assay, *in vivo* tumorigenicity, propidium iodide staining for cell death, analysis of casp3 activity for apoptosis, Matrigel invasion assay, site-directed mutagenesis, luciferase reporter assay, semi-quantitative RT-PCR	miR-27a targets tumor suppressor gene Mcph1, being related with tumorigenic mechanisms, invasions and metastasis	[84]
		miR-196	*Nme4*	Tissue specimens and paired noncancerous matched tissue and plasma from OSCC patients Cell lines: OECM-1 and Fadu OSCC cells and normal oral keratinocytes (NOKs) *In vivo*: nude mice Cell lines: OECM1, SAS, CGHNC8, CGHNC9 and normal keratinocyte cell lines -CGHNK2 and CGHNK4	qRT-PCR, PCR RFLP analysis for rs11614913 genotyping, MTT assay, Transwell migration assay, anchorage-independent colony formation, plasmid construction, Lipofectamine transfection, *in vitro* wound healing assay, Matrigel invasion assay, Western blotting, *in vivo* tumorigenesis, luciferase reporter assay, immunofluorescence staining and confocal microscopy, clinical data analysis	Prognostic marker, overexpressed in plasma and tumor tissue samples. Overexpression was related with a reduced survival and metastatic processes. Regulated cell proliferation, invasion and metastasis	[89,90]
		miR-504	*Foxp1*	OSCC tumor samples from patients Cell line: CA9-22, CAL-27, HSC-3, SAS and TW2.6 *In vivo*: C.B.17-SCID mice	microarray, qRT-PCR, Western blotting, plasmid construction, Lipofectamine transfection, MTT assay, Boyden chamber assays, wound-healing migration assay, animal metastasis experiment, luciferase reporter assay, clinical data analysis	Therapeutic target for reducing invasion and metastasis mechanisms via miR-504/FOXP1 axis	[85]
	Down	miR-17/20a	*Itgb8*	OSCC tumor samples from patients Cell lines: CA9-22, CAL-27, HSC-3, SAS and TW2.6 MS-10	qRT-PCR, Lipofectamine transfection, wound-healing migration assay, luciferase reporter assay, plasmid construction, clinical data analysis	miR-17-92 cluster plays an essential role in inhibiting cell migration. Prognostic marker, miR-17/20a expression decreases with OSCC disease progression.	[93]
		miR-29a	*MMP2*	Cancer tissue and adjacent noncancerous tissue from OSCC patients Cell lines: SCC-4, SCC-9 and SCC-25	qRT-PCR, Western blotting, Lipofectamine transfection, luciferase reporter assay, MTT assay, Transwell invasion assay, flow cytometry	miR-29c mimic have no effect on cell proliferation, but it increase the chemo sensitivity and it decreases the migration of cells	[91]
		miR-124	*Itbg1*	Cell lines: SCC4 and H357	Oligofectamine transfection, luciferase reporter assay, site directed mutagnesis, qRT-PCR, immunoblotting, cell adhesion assay, Transwell chamber assay, Matrigel invasion assay, cell staining with crystal violet	Regulate cell invasion and migration; decrease the adherence to fibronectin only in H357 cells, not in SCC4 cells.	[94]
		miR-138	*RhoC* *Rock2*	Cell lines: UM1, UM2, 1386Ln, 686Ln and primary normal human keratinocytes	transfection with DharmaFECT Transfection Reagent, qRT-PCR, Western blotting, dual luciferase reporter assay, Oris™ cell migration assay kit, Cultrex membrane invasion assay kit, cell stress fiber visualization	miR-138 mimic increase migration capacity; anti-miR-138 activate EMT	[95]
		miR-140-5p	*Adam10*, *ERBB4*	Cell lines: CAL27 and Tca8113	transfection with Turbofect transfection reagent, Western blotting, luciferase reporter assay, cell proliferation assay-CCK8 reagent, Matrigel invasion assay	Transfection with miR-140-inhibited the migration and invasion capacity of Cal27 cells	[92]
Metastasis	Up	miR-146a	*Irak1* *Traf6* *Numb*	OSCC tumor samples and whole blood from patients Cell lines: FaDu, HSC3, OECM-1, SAS, NHOK, 293FT *In vivo*: NOD/SCID mice	qRT-PCR from tissue and plasma, plasmid construction, lentiviral infection, NFκB activity assay, Western blotting, trypan blue exclusion assay, Transwell invasion assay, anchorage-independent growth assay, tumorigenesis, *in vivo* metastasis and experimental therapy	Prognostic marker, overexpression related with unfavorable survival; promote tumorigenesis	[96]
	Down	miR-15b	-	Tissue samples from TSCC patients Cell lines: CAL27 and SCC25 *In vivo*: BALB/c-nu mice	MTT assay, microarray analysis, Lipofectamine transfection, qRT-PCR, Western blotting, modified Boyden chamber assay, immunofluorescence staining, luciferase reporter assay, in-situ hybridization, immunohistochemistry, *in-vivo* metastasis analysis	Regulate chemotherapy induced EMT, dug resistance and metastatic processes	[98]
		miR-99a	*Igfr1R*	Cancer tissue and adjacent noncancerous tissue from OSCC patients Cell lines: CGHNC9, OC3, OEC-M1, TW2.6, FaDu, KB, SCC-4, SCC15, SCC9, SCC25, UT-MUC-1, YD-15, DOK, Tu183, UMSC and HSC3	RT-PCR, immunoblotting, MTT assay, Matrigel invasion assay, *in vivo* lung colonization assay, immunofluorescence, lentiviral infection, luciferase reporter assay, plasmid construction with insulin-like growth factor I receptor, transfection by using Polyjet transfection reagent, cell treatment with 5-Aza-dC (5 μM)	miR-99a act as tumor metastasis suppressor; prognostic marker	[99]
		miR-181a	*Twist1*	Cell lines: CAL27 and SCC15	Lipofectamine transfection, cisplatin chemosensitivity assay, immunofluorescence staining, immunoblotting, RT-PCR, wound healing assay, Transwell invasion assay, dual luciferase reporter assay	Involved in chemoresistance, EMT and metastatic potential *Twist* is a direct target It stimulates E-cadherin and inhibits Vimentin	[100]
		miR-200b	-	Tissue samples from TSCC patients Cell lines: CAL27 and SCC25 *In vivo*: BALB/c-nu mice	MTT assay, microarray analysis, Lipofectamine transfection, qRT-PCR, Western blotting, modified Boyden chamber assay, immunofluorescence staining, luciferase reporter assay, *in-situ* hybridization, immunohistochemistry, *in-vivo* metastasis analysis	Poor prognostic chemoresistance-mediated EMT, invasion and metastasis ↑E-cadherin and ↓Vimentin, N-cadherin and fibronectin	[98]
		miR-491-5p	*Git1*	Cancer tissue and adjacent noncancerous tissue from OSCC patients Cell lines: CGHNC9, SAS, SCC25, OECM-1 and OC-3 *In vivo*: CB17-SCID mice	microarray, plasmid construction, Lipofectamine transfection, immunoblotting, immunohistochemistry, 3′UTR reporter assays, qRT-PCR, falcon cell culture inserts with or without Matrigel invasion assay, *in vivo* lung metastasis assays, immunostaining, immunofluorescence microscopy, Western blotting, Paxillin degradation assays, IHC, FISH, gelatin zymography	Advanced stages It impairs lung metastasis, invasion, focal adhesion and migration through the modulation of MMP2/9 GIT1 is one of its targets	[101]

**Table 3:** The main altered lncRNAs with implication in key biological processes of OSCC.

LncRNA	Expression Level in Tumor Tissue	Biological Role	Target Gene	Role	Molecular Functions	Reference
MEG3	Down	Cell proliferation, cell cycle and apoptosis	*Dnmt3B*	Prognostic marker	sponging, scaffold	[141]
Hotair	Up	Cell proliferation, EMT	*Ezh2*, *E-cadherin*	Diagnostic/prognostic marker	*trans* regulation of gene expression, scaffold	[109,119,120,121,145]
Malat-1	Up	Cell cycle, EMT	*Cks1*, *Wnt*/*β-catenin*	Diagnostic/prognostic	*cis* and *trans* regulation of gene expression, sponging	[120,121,122,146,147]
Ccat2	Up	Cell proliferation	*Wnt*/*β-catenin, Ccnd1*, and *Myc*	Diagnostic/prognostic/therapeutic target	Sponging, scaffold	[10,142,148,149]
Uca1	Up	Cell proliferation, EMT	*Wnt*/*β-catenin*	WNT/β-catenin	Sponging, *cis* regulation of gene expression	[135]
Has2-As1	Up	Hypoxia, EMT	*TGF-α*, *HIF-1α* and *NfKb*	Diagnostic/prognostic	*cis* regulation of gene expression	[143]
Tug1	Up	cell proliferation, apoptosis and invasion, EMT	*Wnt*/*β-catenin*, *cyclin D1*, and *c-Myc*	Therapeutic target	Sponging, decoy	[123]
Hifcar	Up	Hypoxia	*HIF-1α* and *p300*	Prognostic/therapeutic target	Sponging, *cis* regulation of gene expression	[133]
linc-RoR	UP	Stem cells differentiation	*TFs Oct4*, *Nanog*, *Sox4*, *Klf4*, and *c-Myc*	Prognostic/therapeutic target	Sponging, decoys gene-specific histone methylation to promote tumorigenesis	[144,150]
